# Supplementary material for: Transition to Pediatric Practice: A Residency Elective Experience to Prepare Senior Pediatric Residents for General Pediatric Primary Care
Source: MedEdPORTAL. 2016 Nov 22;12:10506. doi: 10.15766/mep_2374-8265.10506 (PMC6440492; doi:10.15766/mep_2374-8265.10506)
Supplement: Supplementary file 1 — A. Transition to Pediatric Practice Curriculum Selective Options.docx B. Sample Individualized Learning Plan for Transition to Practice.docx C. Sample Transition to Practice Schedule.docx D. Coding for Pediatrics Presentation.ppt E. RBRVS Presentation.pdf [file mep-12-10506-s001.zip › _Educational Summary Report_10506.pdf]

MedEdPORTAL®
The Journal of Teaching and Learning Resources

Crossref

Original Publication

OPEN ACCESS

Transition to Pediatric Practice: A Residency Elective Experience to Prepare Senior Pediatric Residents for General Pediatric Primary Care

Maria Kelly, MD, Molly Posa, MD*
*Corresponding author: mollyposa@peds.ufl.edu

Citation: Kelly M, Posa M. Transition to pediatric practice: a residency elective experience to prepare senior pediatric residents for general pediatric primary care. *MedEdPORTAL*. 2016;12:10506. https://doi.org/10.15766/mep_2374-8265.10506

Copyright: © 2016 Kelly and Posa. This is an open-access article distributed under the terms of the Creative Commons Attribution license.

Appendices

A. Transition to Pediatric Practice Curriculum Selective Options.docx
B. Sample Individualized Learning Plan for Transition to Practice.docx
C. Sample Transition to Practice Schedule.docx
D. Coding for Pediatrics Presentation.ppt
E. RBRVS Presentation.pdf

All appendices are peer reviewed as integral parts of the Original Publication.

Abstract

Introduction: Transition to practice (TTP), while impactful in other specialties, has been minimally studied and rarely offered in pediatric training residency programs. This pediatric TTP elective is designed to provide a glimpse into the world of a primary care pediatrician to residents who are interested in pursuing a career in primary care. **Methods:** During this elective residents hone their outpatient diagnostic skills by participating in a variety of clinical patient encounters; this is supplemented with selectives, learner-chosen supplemental educational activities that aim to help fulfill a resident's self-identified learning goals. This TTP experience was developed for third-year pediatric residents who are planning on entering primary care. The course is organized and facilitated by a general pediatric faculty member with an administrative assistant. **Results:** This TTP elective was evaluated highly by pediatric residents; the overall score of its effectiveness, rated by residents who participated in the elective, was 4.5-5.0 out of 5.0. Following completion of this TTP elective, residents demonstrated an overall improvement in outpatient procedural opportunities and self-reported competence for routine outpatient procedures. In addition, residents demonstrated an overall improvement in office-visit billing practices. **Discussion:** The 4-week rotation format maximizes the number of general pediatric outpatient clinical experiences and individualized learning selectives.

Keywords

Resident Education, Transition to Practice, General Pediatrics, Outpatient Pediatrics, Autonomous Patient Care, Pediatric Residency Training

Educational Objectives

By the end of this elective, the resident will be able to:

1. Gain insight into real-world outpatient primary care pediatrics.
2. Improve proficiency in evaluating and treating patients with minimal physician supervision.
3. Develop competence in pediatric billing and coding.
4. Develop a professional curriculum vitae and explore the job search process.

Introduction

Transition to practice (TTP) education is defined as an experiential opportunity for mentored practice experiences.[1] Evidence suggests that residents have a variety of affective reactions when faced with their transition to independent practice following residency completion.[2] TTP experiences have slowly evolved since Accreditation Council for Graduate Medical Education (ACGME) duty hour rules created limitations on residents' abilities to experience comprehensive autonomous care. It is also known that an academic-based continuity clinic experience is not comparable to real-world clinical care.[3] TTP educational electives during residency training have been created with positive resident experiences in multiple different medical fields, including family medicine,[4] neurological surgery,[1] and anesthesiology.[5] Despite this, TTP experiences and studies of transition to independent practice in pediatrics are limited.

In 2011, a group of physician educators from the University of Florida distributed a national survey regarding TTP educational experiences for pediatric residents through the Association of Pediatric Program Directors national LISTSERV. This survey revealed that although 50% of pediatric residents will choose positions in pediatric primary care following residency training, less than half of pediatric residency programs were using a formal residency experience/elective to facilitate the transition. Of those programs with developed pediatric electives, most concentrated on clinical experiences, practice management, and billing/coding. These findings were presented at the 2012 Pediatric Academic Societies' national meeting. With increased pressures on residency programs, it is imperative that pediatric residency programs train residents in a formal manner for this important transition and continue to expand these educational experiences for future pediatric primary care physicians.

Following this background research, as well as further informal feedback and encouragement at national pediatric meetings, researchers were encouraged to create and implement a formal TTP curriculum. With the help of general pediatricians and educators, a TTP curriculum was created and introduced at the University of Florida in 2012 for senior pediatric residents preparing for general pediatric outpatient practice. The rotation was positively received and has subsequently evolved based on faculty and resident feedback through both formal and informal evaluations.

Methods

This TTP or private practice elective is designed to give pediatric residents who are interested in pursuing a career in primary care a glimpse into the world of pediatric primary care after residency. The target audience for this rotation is third-year pediatric residents who plan on entering the field of primary care pediatrics after graduation. The only prerequisite to the rotation is that the resident must complete two core electives before being permitted to participate in the pediatric TTP elective. This assures there is no compromise in the resident's ability to meet graduation requirements for ACGME-required rotations as well as maximizing the educational value of a pseudo-independent clinical practice.

Logistics

Prior to the start of each rotation, the resident is asked to review the TTP syllabus and create an individualized learning plan (ILP) that includes at least four to five selectives to explore the resident's postresidency goals. All the possible selective choices are listed in the TTP curriculum selective option document (Appendix A).

A requirement of the TTP elective is additional clinical outpatient sessions, but there is unscheduled time set aside for the resident's selectives. The selectives have been broken down into clinical development, administration, community partnering, academics, and mentoring. It is also encouraged that pediatric residents should speak to other residents who have previously participated in the rotation or to the elective director to help them choose specific selectives that would be the most beneficial for their specific goals.

Preparation

Residents create their ILP, which includes their selectives and self-identified learning goals for the rotation. A sample resident ILP has been included for reference (Appendix B). The resident is also asked to alert the elective administrators to vacation dates, backup dates, and any other prescheduled events or obligations that must be taken into consideration when creating the resident's individualized schedule. The ILP is to be submitted to the elective director no later than 2 weeks prior to the start of the rotation, ensuring that the elective director and resident administrator have ample time to coordinate the resident's individualized selectives in conjunction with the resident's required additional clinical sessions.

An individualized schedule (Appendix C) that includes the required clinical experiences and as many selectives as possible without compromising the resident's clinical experience is then constructed. As faculty elective rotation coordinators, we at times insert high-yield selectives into a resident's schedule if

openings exist, even if not chosen by the resident, particularly the billing/coding experience. This generally results in four to five clinical sessions per week and four to five selectives during the monthlong rotation. Call, weeknight, and weekend responsibilities are tailored to the goals and objectives expressed by the resident and may include nighttime newborn nursery call, newborn nursery weekend rounding coverage, pediatric after-hours acute experience, and/or Saturday morning acute clinic experience. This schedule is then sent to the participating resident, chief residents, clinical nursing supervisors, clinic site supervisors, clinic physician attendings, and selective facilitators prior to the resident's first day on the rotation.

For the billing/coding selective, the resident reviews the educational PowerPoints (Appendices D & E) created by a divisional and nationally recognized expert coder.

Limitations
Due to clinic space limitation and other educational commitments, this pediatric TTP elective accepts only a single third-year resident per month, with a minimum of 2 weeks of participation required in a 4-week period.

Formative feedback regarding the resident's performance is completed halfway through the monthlong rotation by the faculty elective director. Formal evaluations are completed at the end of the rotation by all faculty members who worked with the resident in any clinical setting. At the end of the rotation, the faculty elective director and resident meet to discuss the rotation, and the resident also completes a formal evaluation of the rotation per ACGME requirements.

Results

On average, 50% of the third-year pediatric residents (and 100% of those choosing a primary care career following graduation) at our pediatric residency training program elected to participate in the TTP elective, which is approximately six to eight residents a year. Although this elective was selected primarily by third-year senior residents entering the field of general pediatrics (90% of participants), some residents entering pediatric hospital medicine and/or subspecialty fellowships also participated in this elective (10%). The elective was evaluated highly by pediatric residents and has consistently received very positive feedback. Since creation, the TTP rotation has received average scores of 4.5-5.0 out of 5.0 by residents evaluating the elective's effectiveness in regard to preparing them for independent practice after residency. Comments written by residents regarding the rotation included the following:

- "Great experience for preparing to go into private practice."
- "Excellent elective; the best I've ever done. The TTP elective is so incredibly organized, well run, and was incredibly helpful when preparing to enter the real world. It was a perfect balance of all things important during a difficult transition period and I loved that we had input into what we spent our time doing that month."
- "It is a must for anyone even considering general pediatrics. I learned so much about a general practice management that I don't think I would have been introduced to otherwise."
- "One of the best rotations our residency has to offer."
- "The sessions on coding, billing, and practice management were more insightful than I ever imagined! Overall a very useful, very well structured rotation."
- "Added to my clinical experience. My favorite part about TTP was acting as a pretending in the outpatient clinics. It was a true glimpse into being an independent practitioner and was something unheard of on other rotation and electives."
- "Great variety of options to individualize the rotations. Helpful and flexible in meeting each resident's needs and what they desire to get out of the rotation."

The creators of this course, in conjunction with participating faculty members, have investigated the effectiveness of the TTP elective and presented their findings nationally following a review of the first 2 years of data. Investigations of this course, specifically, the effectiveness of billing/coding education and procedural competence following the rotation, have been presented at the Pediatric Academic Sciences' national meetings twice. It was found that following completion of the TTP elective, residents demonstrated an overall improvement in outpatient procedural opportunities and self-reported competence for routine outpatient procedures (Table 1). In addition, residents demonstrated an overall improvement in visit coding/billing practices. Specific results included that during the past 3 academic years, 50% of residents (N = 24, out of a possible 48) participated in the TTP elective. A comparison of pre- and postcoding assessments among the participating TTP elective residents revealed a significant difference between pre and post scores ($p < .05$) relating to selection of appropriate vaccination administration codes, use of modifiers, and level of service. During the preassessment, residents underbilled 37% of visits and overbilled 4% of visits, while postassessment billing errors decreased to 7% and 2%, respectively (Table 2). Overall, residents performed a total of 56 procedures during the elective, with any resident performing between zero and nine procedures. Improved self-perceived procedural competence ($p < .05$) was achieved for circumcision, cryotherapy, urine catheterization, and intramuscular injection (Table 1).

Table 1. Self-Reported Procedural Competence[a]

Procedure Assessed	*N*	Pre-TTP	Post-TTP	*p*
Circumcision	18	4	4.7	<.01
Lesion cryotherapy	10	3.9	4.9	<.01
Urine catheterization	11	2.1	4.4	<.01
Intramuscular injection	16	2.3	4.7	<.01
Frenotomy	5	4	4.3	NS
Incision and drainage	6	4.8	5	NS
Tympanogram	7	2.3	3.7	NS
Pelvic examination	4	5	5	NS
Punch biopsy	1	1	4	NS
Capillary draw	1	1	4	NS

Abbreviations: NS, not significant; TTP, transition to practice.
[a]Rated on a Likert scale where 1 = *no experience* and 5 = *performed independently*.

Table 2. Pre- and Postelective Coding Assessments

Coding Parameter Assessed	Pre-TTP (%)[a]	Post-TTP (%)[b]	*p*
Level of service correctly coded	150 (60%)	222 (91%)	<.05
Level of service undercoded	89 (36%)	17 (7%)	<.05
Level of service overcoded	10 (4%)	4 (2%)	<.05
Modifier correct	45 (18%)	211 (87%)	<.01
Vaccine administration code correct	102 (41%)	219 (90%)	<.01
Procedural CPT code correct	227 (91%)	238 (98%)	NS

Abbreviations: CPT, current procedural terminology; NS, not significant; TTP, transition to practice.
[a]N = 249.
[b]N = 243.

Discussion

The creation of the TTP elective was based on a residency program needs assessment and a national survey, which verified this need on a national level. The original curriculum has been adapted over time to refine and introduce new selective opportunities based on faculty availability and expertise and to meet individual resident needs. It is recognized that some of these selectives may not be available at all programs and/or may require faculty development. Aspects of this elective experience have been formally evaluated; following completion of the TTP elective, residents demonstrated an overall improvement of billing/coding practices and competence in common outpatient pediatric procedures. These results have been presented on a national level twice and have received positive feedback with encouragement for national dissemination of our curriculum. On average, 50% of graduating pediatric residents enter general pediatric practice; therefore, the TTP curriculum can be a valuable method for other residency programs to maximize pediatric resident preparedness for transition into pediatric primary care.

We do recognize a few limitations for this elective. Successful incorporation of the TTP experience is dependent on many factors. Pediatric residency programs should have a strong general pediatric division

that will support this experience within their outpatient clinics. There must be an already existent infrastructure to introduce an additional third-year resident into the outpatient pediatric clinics. Required clinic infrastructure would include, at a minimum, an additional independent resident schedule, two to three patient rooms, nursing support, administrative support (to create patients schedules and grids), and a supervising faculty member who feels comfortable allowing the resident a pseudo-independent level of autonomy. In addition, the resident should have a patient panel consistent with a rigorous, real-life outpatient clinic schedule. The residency program must also have faculty members and ancillary staff with experience or familiarity in the above-mentioned selective areas.

Each institution will need to explore its ability to offer these selective opportunities. We have found we need to review/augment our selective list yearly, secondary to faculty turnover and availability. On a positive note, we have also added selectives based on incoming faculty with expertise or experience in areas not previously offered. For this reason, the curriculum needs to be reviewed on a yearly basis for changes and additions. For successful elective creation, it is also encouraged that residency programs explore a relationship with private pediatric practitioners and institutional resources within their geographic area as resources for many of the selectives (billing/compliance, private practice providers, social work, medical daycare, etc.). It is essential to have community and institutional outreach and participation for many of the selective experiences to be effective and a positive experience for the participating residents. Finally, creation of the monthly schedule can be cumbersome and time-consuming. For this reason, the faculty elective director should have administrative support to organize the rotation, create the schedule, and communicate details to the resident and participating faculty members. It is also imperative that both faculty and resident participants evaluate the elective so it can be adapted to optimize maximal positive impact for all participants.

Maria Kelly, MD: Associate Professor, Department of Pediatrics, University of Florida College of Medicine

Molly Posa, MD: Assistant Professor, Department of Pediatrics, University of Florida College of Medicine

Disclosures
None to report.

Funding/Support
None to report.

Ethical Approval
This publication contains data obtained from human subjects and received ethical approval.

References

1. Murad GJ, Lister JR, Friedman WA, Lombard G. Enhancing competence in graduates through a transition to practice program in neurological surgery. *Clin Neurosurg*. 2010;57:141-144.
2. Brandt MT. Transitioning from residency to private practice. *Oral Maxillofac Surg Clin North Am.* 2008;20(1):1-9. http://dx.doi.org/10.1016/j.coms.2007.09.002
3. Serwint JR, Thoma KA, Dabrow SM, et al. Comparing patients seen in pediatric resident continuity clinics and National Ambulatory Medical Care Survey practices: a study from the Continuity Research Network. *Pediatrics.* 2006;118(3):e849-e858. http://dx.doi.org/10.1542/peds.2006-0422
4. Tamblyn R, Abrahamowicz M, Dauphinee D, et al. Effect of a community oriented problem based learning curriculum on quality of primary care delivered by graduates: historical cohort comparison study. *BMJ.* 2005;331(7523):1002. http://dx.doi.org/10.1136/bmj.38636.582546.7C
5. Holak EJ, Kaslow O, Pagel PS. Facilitating the transition to practice: a weekend retreat curriculum for business-of-medicine education of United States anesthesiology residents. *J Anesth.* 2010;24(5):807-810. http://dx.doi.org/10.1007/s00540-010-0973-7

Received: July 22, 2016 | **Accepted:** November 7, 2016 | **Published:** November 22, 2016
