# Supplementary material for: Transition to Pediatric Practice: A Residency Elective Experience to Prepare Senior Pediatric Residents for General Pediatric Primary Care
Source: MedEdPORTAL. 2016 Nov 22;12:10506. doi: 10.15766/mep_2374-8265.10506 (PMC6440492; doi:10.15766/mep_2374-8265.10506)
Supplement: Supplementary file 1 — A. Transition to Pediatric Practice Curriculum Selective Options.docx B. Sample Individualized Learning Plan for Transition to Practice.docx C. Sample Transition to Practice Schedule.docx D. Coding for Pediatrics Presentation.ppt E. RBRVS Presentation.pdf [file mep-12-10506-s001.zip › A. Transition to Pediatric Practice Curriculum Selective Options.docx]

**The “Selectives” Menu: Customizing the TTP Elective**

The “Selectives” menu is described in more detail below. Each resident is asked to identify specific “selectives” from the menu below to help address their Individualized Learning Plan (ILP) goals for this rotation. Although the clinical outpatient sessions are required, there is unscheduled time set aside for the resident’s “selectives”. Residents are asked to list the “selectives” that they are interested in participating in during their TTP elective rotation (5 “selectives” minimum or 1-2 “selectives”/week). The “Selectives” are broken down into the following categories:

- 1. Clinical development (complex care coordination, adolescent and young adult care, ADHD and developmental clinic, infant and toddler dental clinic, breastfeeding medicine clinic, community private practice clinic experience)
  2. Administration (billing and coding, staffing, documentation, administrative responsibility, national pediatric organizations)
  3. Community partnering (communication with other physicians, communication with patients, parents and caretakers, resource networking, advocacy)
  4. Academics (resident and medical student supervision, formal didactics, pediatric product knowledge and application, clinical pathway/protocol or quality improvement, research and scholarship)
  5. Mentoring (lifelong learning plan, giving and receiving feedback, work-life balance, starting the job search, writing a CV and cover letter, financial planning experience)

**Clinical Development (Patient Care, Clinical Practice, Practice-Based Learning and Improvement)**

Improve patient care skills for independent practice in the outpatient pediatric clinical setting with attention to following best practices guidelines.

1. Autonomous Patient Care (*REQUIRED COMPONENT OF THE TTP ROTATION)
   1. The resident will have the opportunity to see scheduled patients independently on a predetermined scheduling template. This will include a variety of patient encounters that can be tailored to a resident’s request. Examples of encounters include urgent (sick) visits, return visits (including follow-ups, ADHD, obesity, asthma), health supervision visits, supervision of nurse only encounters, office-based procedures. These patients will then be presented and discussed with the supervising attending physician following the patient encounter or at the end of the clinic session. This gives the resident the unique opportunity to have an in-depth discussion with the faculty member about how to approach a variety of patient presentations with regards to clinical care, coding and billing and arranging follow-up or subspecialty referrals. The location of the selected clinic can be based on the resident’s requests (private practice, academic-based, inner-city, ADHD clinic, adolescent clinic). The predetermined scheduling template should resemble a more typical general practice clinic session (equal distribution of urgent, well and return visits), which differs from the resident continuity experience where a majority of visits are health supervision visits at our institution.
2. Complex Care Coordination
   1. The resident will spend time working with the Pediatric Care Coordination Program (PCCP) team to better understand the specialized outpatient needs of children with complex medical conditions.
      1. The resident will participate in all aspects of complex medical care including care coordination with specialty and referral services, patient care (through patient calls and clinical encounters) and learn specialized coding and billing issues as they apply to this subset of patients.
      2. The resident will gain insight and knowledge regarding specialized medical equipment (home-based mechanical ventilation, feeding pumps, wheelchairs), medical needs (G-tube, tracheostomy care, central access ports) and how to access home health nursing as it applies in the outpatient setting.
      3. The resident will learn how to assess and manage the multiple medical difficulties of a medically complex patient and better understand the medical home model.
   2. The resident will visit the Prescribed Pediatric Extended Care (PPEC) center, a medical daycare, to better understand what a medical daycare entails as it applies to patients with complex medical needs. This includes the scope of medical care offered, how to refer a patient and documentation required from the primary care physician for maintenance of care.
   3. The resident will read the following articles prior to participation in the above mentioned activities:
      1. Juhlmann AF. Take the little steps: providing complex care. *Pediatr Ann*. 2010;39(4): 248-53.
      2. Agrawal R. Complex Care. *Pediatr Ann*. 2010;39(4): 186.
      3. Shulman ST. Complex Care is Complicated! *Pediatr Ann*. 2010;39(4): 183-4.
      4. Mukherjee S, Coha T. Common Skin Problems in Children with Special Healthcare Needs. *Pediatr Ann*. 2010; 39(4): 206-15.
      5. Srivastava R, Jackson WD. Dysphagia and Gastroesophageal Reflux Disease: Dilemmas in Diagnosing and Management in Children with Neurological Impairment. *Pediatr Ann*. 2010; 39(4): 225-231.
      6. Hauer J. Identifying and Managing Source of Pain and Distress in Children with Neurological Impairment. *Pediatr Ann*. 2010; 39(4): 198-205.
3. Adolescent and Young Adult Care
   1. The resident will spend time working with adolescent faculty, within the Adolescent and Young Adult Clinic, to improve their clinical and diagnostic skills in the well and sick management of adolescent patients. The resident will have their own panel of adolescent patients and will present and discuss the encounters with the supervising attending physician following the patient encounter or at the end of the clinic session. This gives the resident the unique opportunity to have an in-depth discussion with the faculty member about how to approach a variety of adolescent patient presentations with regards to clinical care, coding and billing and arranging follow-up or subspecialty referrals.
   2. The resident will perform common adolescent procedures under the supervision of an attending in the adolescent clinic (including but not limited to pelvic/bimanual exams, verruca removal, venipuncture and intramuscular injections).
   3. The resident will review adolescent topics in an informal manner with the supervising faulty member as related to patient care (high risk sexual activity, menstrual irregularities, acne, OCPs, depression, illicit drug use, school failure, family conflict, etc).
4. ADHD and Developmental Clinic
   1. The resident will spend time working in the ADHD/Development clinic, to improve their clinical and diagnostic skills in these areas. The resident will have their own panel of patients and will present and discuss the encounters with the supervising attending physician following the patient encounter or at the end of the clinic session. This gives the resident the unique opportunity to have an in-depth discussion with the faculty member about how to approach a variety of clinical presentations with regards to clinical care, coding and billing and arranging follow-up/further educational or behavioral testing.
   2. The resident will review developmental and ADHD topics in an informal manner with the attending as related to patient care (developmental delay screening, neuropsychoeducational testing, ADHD medications, ADHD medication side effects, comorbid conditions, school failure, etc).
5. Infant and Toddler Dental Clinic
   1. The resident will spend time working with an academic-based pediatric dentist in the university affiliated pediatric dental clinic.
   2. The resident will gain a general understanding and knowledge base regarding normal dental health and common dental diseases, including recognizing early dental decay, dental caries and abnormal dentition and be able to provide appropriate anticipatory guidance to parents regarding oral health.
   3. The resident will become competent in the application of topical fluoride.
6. Breastfeeding Medicine Clinic
   1. The resident will spend time working with a certified lactation consultant in an outpatient clinical setting.
   2. The resident will gain a general understanding and knowledge base regarding the role of a lactation consultant, ability to assess adequacy of breastfeeding and the ability to perform anticipatory guidance for common breastfeeding problems.
   3. The resident will have the opportunity to perform frenotomies under supervision.
7. Community Private Practice Clinic Experience
   1. The resident will spend time working with a community pediatrician to better understand clinical decision making in a busy outpatient private practice. This will give the resident the unique opportunity to have an in depth discussion with the community pediatrician about how to approach a variety of patient presentations with regards to clinical care, coding and billing and arranging follow-up or specialty needs in a non-academic environment.
   2. The resident will meet with their office staff and practice manager at the non-academic pediatric site to discuss their processes of billing, referrals, appointments and scheduling in a private practice clinic.

**Administration (Systems-Based Practice)**

Increase experience and familiarity with being an integral part of not only practice policy, but also hospital and state-wide infrastructure. The resident will be given information that includes documentation requirements, billing and coding examples and definitions designed to help clarify the terminology of billing and coding.

1. Billing and Coding
   1. The resident will review educational PowerPoints on billing/coding created by a divisional and nationally recognized expert coder. (Appendices D and E)
   2. The resident will practice billing and coding for their clinic patients with real-time feedback by clinic attending physicians.
   3. The resident will meet with a Compliance Officer from the Office of Physician Billing Compliance to review 10 patient encounters from a billing perspective.
   4. The resident will review the relevant articles included in their Transition to Practice binder.
      1. Jensen, P. Coding “Routine” Office Visits: 99213 or 99214. *Family Practice Management*. 2005; 12(8):52-57.
      2. Hill, E. How to Get All the 99214s You Deserve. *Family Practice Management*. 2003; 10(9):31-36.
      3. Hill, E. Understanding When to Use the New Patient E/M Codes. *Family Practice Management*. 2003; 10(8):33-36.
      4. Weida, T and O’Gurek, D. Coding from the Bottom Up. *Family Practice Management*. 2008; 15(9):22-25.
      5. Schuman, A. Level 4 office-visit coding. *Contemporary Pediatrics*. 2013; 30(2):37-41.
      6. Traisman, E. Pediatric Coding: What You Need to Know to Enhance Your Clinical Practice’s Reimbursement Process. *Pediatric Annals*. 2010; 39(6):362-66.
2. Staffing
   1. The resident will meet with the nursing and practice manager at one of the outpatient clinics to learn about clinic staffing needs and coordination to maximize clinical operations while managing operational expenses. Ideally this experience should occur prior to the “Community Private Practice Clinic Experience” selective, if chosen.
      1. The resident will meet with the nursing supervisor to understand different qualifications and roles of nurses (LPN, RN) and assistants (MA, MT) in clinic. The resident will discuss how the nursing supervisor creates the weekly nursing schedule. They will also review vaccine storage, administration and documentation protocols in the clinic; this includes reviewing the *Vaccine for Children* protocols and requirements.
      2. The resident will meet with the practice manager to understand their role in clinic management and administration. This includes managing costs, understanding cost per visit, reimbursement requirements from various insurers (medicaid versus private insurers), front desk staffing, clerical roles and design of patient flow through clinic.
3. Documentation
   1. The resident will attend a session with a Compliance Officer from the Office of Physician Billing Compliance to better understand documentation practices expected at a resident and an attending level. The resident will bring a copy of 10 patient encounters they completed documentation on during the TTP rotation. They will discuss required documentation in regards to billing and review the billing and coding of these encounters for discrepancies (i.e. over or under billing) or missed opportunities for billings services (i.e. remembering to bill for procedures, developmental screening and counseling for vaccine administration).
   2. The resident will receive direct feedback by outpatient faculty members specifically focusing on resident outpatient documentation skills relating to the perspectives of billing, legal and diagnosis documentation minimums.
   3. The resident will complete the yearly compliance training required of faculty.
   4. The resident will observe a meeting of the Billing and Compliance committee at the University of Florida.
4. Administrative Responsibility
   1. The resident will participate in monthly General Pediatrics divisional meetings.
   2. The resident will participate in a monthly operational meeting at one of the pediatric outpatient clinics.
5. National Pediatric Organizations and Role in Outpatient Pediatric Medicine
   1. The resident will receive an introduction to the various roles of the national societies in pediatric outpatient medicine.
      1. The resident will locate the homepages of the following national organizations and familiarize themselves with their mission statements and the content and resources on the website that are applicable to pediatric primary care. The resident will send applicable links to the elective director.
         - APA
         - PAS
         - AAP
   2. The resident will develop an understanding of current issues in healthcare policy and specific pediatric programs, including CMS, ICHIP, Title 19 and Title 21.
      1. The resident will meet with departmental leaders in advocacy and healthcare policy to discuss their current roles with regional and national organizations to promote healthcare policy for pediatric patients.
      2. The resident will attend a local, regional or national legislative session relating to health policy if possible.
      3. The resident will identify resources (newsletters, listserves, journals, etc) that focus on healthcare policy and serve to update community pediatricians regarding their services.

Community Partnering (Interpersonal and Communication Skills)

Learn the nuances of caring for fellow pediatricians’ patients safely and effectively, establishing networks of communication and enhancing patient and physician education.

1. Communication with other physicians
   1. The resident will observe and then practice phone communication between primary care and after-hours physicians, transition of patients to the emergency room or inpatient setting and/or obtain recommendations from subspecialists for a complicated patient.
      1. The resident will participate in these transitions and be directly observed by faculty via speaker phone (or 3-way calling).
2. Communication with patients, parents, and/or caretakers
   1. The resident will practice phone triage with daily incoming patient calls under direct observation of the triage nurse and provide proper documentation in the patient’s legal medical record discussing recommendations.
      1. The resident will participate by direct observation via speaker phone (or 3-way calling) and receive real-time feedback from the supervising triage nurse.
   2. The resident will contact a patient, parent or caretaker regarding lab or radiologic results.
      1. The resident will participate by direct observation by attending via speaker phone (or 3-way calling) and receive real-time feedback from the observing attending regarding the phone call discussion.
   3. The resident will observe and then practice communication styles and techniques for patient encounters (including discussion of history and anticipatory guidance) in an efficient, yet thorough, manner without compromising patient care.
      1. The resident will participate by direct observation of resident-patient encounters with real time feedback from participating pediatric faculty (structured clinical observation).
3. Resource Networking
   1. The resident will spend time with an outpatient clinic social worker, nutritionist at Women Children and Infant (WIC) Clinic, physical therapist, occupational therapist or speech and language pathologist to enhance their knowledge of outpatient services available for patients in the outpatient setting. The resident will help identify the resources that are most beneficial for their patients’ needs.
   2. The resident will meet with a referral clerk at an outpatient clinic to understand the referral process and how information is processed, communicated and transferred.
   3. The resident will work at the centralized access center to better understand how patient calls are received and handled. This will give them an improved perception of the patient/parental experience when calling a pediatrician’s office.
   4. The resident will spend time with the hospital transfer center to better understand the transfer of patients from the outpatient clinic into the inpatient setting.
4. Advocacy
   1. The resident will advocate for patients with socioeconomic struggles in the outpatient hospital-based practice settings by spending a clinic session focusing on obtaining structured, detailed social and medical home histories.
   2. The resident will meet with the outpatient social worker to explore social and economic challenges and learn about community resources available for families.
   3. The resident will conduct a home visit on one of their patients to better understand challenges and complexities they face in their daily lives and the impact this may have on their health and well-being.

Academics (Medical Knowledge)

Stylize skills within the academic and clinical teaching settings, incorporating evidence-based medicine and real-time knowledge translation.

1. Resident and Medical Student Supervision
   1. The resident will learn to provide at least a single teaching point on every patient they see and recognize the diverse learning styles and medical knowledge understanding of those they are supervising.
      1. Demonstrate this skill with attending physicians, medical students and shadowees while in the clinical setting.
   2. The resident will familiarize themselves and utilize unique teaching skills and methodologies that can be useful in the fast-paced, high turnover outpatient clinical setting.
   3. The resident will spend a clinic session working as a “pretending” for medical students and/or junior residents where they will supervise their clinical care while optimizing opportunities to practice the efficient delivery of teaching points under the supervision of a faculty member.
   4. The resident will read the following articles:
      1. Alevi AM, Lane JL. Microskills in Office Teaching. *Pediatr Ann*. 2010; 39(2):72-7.
      2. Nagappan S, Doyne EO, Roberts K, Dewitt TG. Pediatric Education in Office Settings. *Pediatr Ann*. 2010; 39(2): 67-71.
2. Formal Didactics
   1. The resident will create and present a 30 minute lecture to the medical student(s) on the pediatric clerkship regarding a general outpatient pediatric topic identified as a learning objective in the medical student syllabus.
   2. The resident will receive feedback from a faculty member with experience in medical education on their educational lecture.
3. Pediatric Product Knowledge and Application
   1. The resident will become familiar with the quantity and quality of a multitude of different pediatric products available to parents including formula, bottles, infant supplies, medications, car seats and foods to build a better foundation for parental education during patient encounters.
   2. The resident will spend a session with a pediatric faculty member at a retail outlet store discussing and comparing a multitude of these items and their applicability in different clinical scenarios.
4. Clinical Pathway/Protocol or Quality Improvement
   1. The resident will research and build patient encounter templates or re-evaluate established pathways for common outpatient disease processes or patient presentations.
   2. The resident will develop or evaluate an established clinical protocol for common outpatient disease process using best practice guidelines.
   3. The resident will create a quality improvement project in the outpatient setting and implement it under the guidance of a faculty mentor.
5. Research and Scholarship in the Pediatric Outpatient Setting
   1. The resident will become familiar with research, scholarship opportunities and obstacles/challenges to performing research in the outpatient clinical setting.
   2. The resident will meet with a research faculty member who actively conducts research in the general outpatient setting and discuss the process for development of clinic-based investigations.
   3. The resident will work with a faculty member to create a scholarly project or become involved in an ongoing project. This may include a case-report, community resource article or quality improvement project.
   4. The resident will participate in the monthly divisional research meeting.

Mentoring (Professionalism)

Capitalize on working with multiple outpatient pediatric faculty members to discuss career development.

1. Discuss the importance of developing a lifelong learning plan.
   1. The resident will create an individualized learning plan with the understanding it can be adapted over time.
   2. The resident will learn tips for a rewarding career as a student of medicine.
   3. The resident will meet with an experienced pediatric faculty member to review and discuss the resident’s lifelong learning plan and how it can be adapted to meet requirements of the American Board of Pediatrics Maintenance of Certification process.
2. Giving and Receiving Feedback
   1. The resident will meet with a faculty mentor involved in education and discuss the following topics:
      1. What is formative feedback and why is it important?
      2. How can you give and receive formative feedback from your superiors and your peers?
      3. How to be an active participant in the evaluation process?
      4. Read the following article prior to this session:
         1. Burgess A, Mellis C. Feedback and assessment for clinical placements: achieving the right balance. *Adv Med Educ Pract*. 2015; 6:373-381.
3. Work Life Balance
   1. The resident will meet with various academic and/or community pediatricians to discuss their successes and struggles in balancing the pressures of an academic career, administrative responsibilities, patient care and home life.
   2. The resident will construct a written work-life balance commitment plan individualized to their needs.
4. Starting the Job search
   1. The resident will meet with a faculty member and/or private practice pediatrician to discuss the following topics:
      1. Academic versus community practice setting: Discussing the differences
      2. Finding and critiquing practice opportunities
      3. Contract negotiations
      4. Understanding malpractice in different clinical environments
   2. The resident will meet with an experienced pediatric faculty member involved in the provider recruitment process to discuss the job search and interview process.
   3. The resident will read the following articles:
      1. American Academy of Pediatrics *Launching Your Career in Pediatrics*.
      2. Beach R. Interviewing 101. *Family Practice Management*. 2001; 8(1):38-40.
   4. The resident will participate in a mock interview with a member of the divisional hiring committee.
5. Writing a Curriculum Vitae (CV) and Cover Letter
   1. The resident will explore the AAMC website to review how to write a CV at [https://www.aamc.org/members/gfa/faculty_vitae/150034/preparing_your_curriculum_vitae.html](https://www.aamc.org/members/gfa/faculty_vitae/150034/preparing_your_curriculum_vitae.html)​
   2. The resident will write and/or update their CV and submit their CV for review to the faculty elective director.
   3. The resident will review how to write a cover letter on the American Academy of Pediatrics *Launching Your Career in Pediatrics* website.
   4. The resident will write a sample cover letter and submit for review to the faculty elective director.
6. Financial Planning Experience
   1. The resident will meet with a financial advisor to discuss wealth management, including retirement funds, paying off educational loans, disability insurance and other topics of interest to the individual resident.
