# Supplementary material for: Transition to Pediatric Practice: A Residency Elective Experience to Prepare Senior Pediatric Residents for General Pediatric Primary Care
Source: MedEdPORTAL. 2016 Nov 22;12:10506. doi: 10.15766/mep_2374-8265.10506 (PMC6440492; doi:10.15766/mep_2374-8265.10506)
Supplement: Supplementary file 1 — A. Transition to Pediatric Practice Curriculum Selective Options.docx B. Sample Individualized Learning Plan for Transition to Practice.docx C. Sample Transition to Practice Schedule.docx D. Coding for Pediatrics Presentation.ppt E. RBRVS Presentation.pdf [file mep-12-10506-s001.zip › B. Sample Individualized Learning Plan for Transition to Practice.docx]

Sample ILP for TTP

I’ve looked at the goals/objectives of the Transition to Practice month and here is what I am looking forward to learning this month and how I hope to accomplish those goals. I also want to mention I do have vacation on 4/15-4/17 and would like these days free from activities. I also am on back-up the week of 4/7 just in case that needs to be taken into consideration. I want to let you know I envision myself working in a pediatric office in Denver, Colorado. I will plan on working with both medical students and residents intermittently. I also may be invited to provide lectures to the residency program on general pediatric topics.

1. Goal- Improve my clinical skills and understanding of a real world outpatient pediatric clinic.

Reason- Working in a real outpatient environment will likely be different from my continuity clinic experience. For that reason, I would like to experience a more “real-world” type schedule and better understand the practices of a clinic. I would like to work on my efficiency during my clinical visits.

Selectives:

- - 1. Autonomous Patient Care that includes primarily urgent, return and well child visits.
    2. Adolescent and Young Adult Care
    3. Billing and Coding
    4. Staffing
    5. Documentation
    6. Pediatric Product Knowledge and Application
    7. Community Private Practice Experience

1. Goal- Improve my clinical skills and understanding of breastfeeding problems and how to manage them.

Reason- While working in the newborn nursery, I did not spend much time with a lactation consultant. Through my continuity and acute experiences, I realize breastfeeding issues are a common complaint and concern.

Selectives:

- - 1. Breastfeeding clinic

1. Goal- Improve my ability to teach in an outpatient setting.

Reason- I plan to eventually have learners in my new practice- either medical students or residents. For that reason, I would like to practice teaching in a busy outpatient setting and learn tips.

Selectives:

- - 1. Giving and Receiving Feedback
    2. Formal Didactics
    3. Resident and Medical Student Supervision

1. Goal- Work on a quality improvement project in the outpatient setting

Reason- I’d like to improve my Anticipatory Guidance and really model it after Bright Futures. I have done this to some extent but have felt constrained for time to be able to go through the recommendations. What I would like to do is make templates in Epic that re-enforce the Bright Futures principles and can make them more readily accessible for other resident and attendings.

Selectives:

- - 1. Clinical Pathway/Protocol or Quality Improvement
