# Supplementary material for: Transition to Pediatric Practice: A Residency Elective Experience to Prepare Senior Pediatric Residents for General Pediatric Primary Care
Source: MedEdPORTAL. 2016 Nov 22;12:10506. doi: 10.15766/mep_2374-8265.10506 (PMC6440492; doi:10.15766/mep_2374-8265.10506)
Supplement: Supplementary file 1 — A. Transition to Pediatric Practice Curriculum Selective Options.docx B. Sample Individualized Learning Plan for Transition to Practice.docx C. Sample Transition to Practice Schedule.docx D. Coding for Pediatrics Presentation.ppt E. RBRVS Presentation.pdf [file mep-12-10506-s001.zip › C. Sample Transition to Practice Schedule.docx]

**“Transition To Practice” – (1/11-2/5)**

| ` | Mon AM  8a-12p | Mon PM  1p-5p | | Tues AM  8a-12p | Tues PM  1p-5p | Wed AM  8a-12p | Wed PM  1p-5p | Thur AM  8a-12p | Thur PM  1p-5p | Fri AM  8a-12p | Fri PM  1p-5p |
| --- | --- | --- | --- | --- | --- | --- | --- | --- | --- | --- | --- |
| 1/11-1/15 | **Meet with faculty coordinator to review TTP packet and get started.**  **8:30am** | **Patient Panel**  **Magnolia Parke Clinic** | | **Patient Panel**  **Magnolia Parke**  **Clinic** | **RESIDENT**  **LECTURES** | **PCCP Clinic- Care of the chronically ill child**  **CMS Clinic** | **Patient Panel**  **Tower Square**  **Clinic** | **Continuity Clinic**  **CMS Clinic** | **Adolescent Clinic**  **CMS**  **Clinic** | **Private Practice Shadow** | **Private Practice Shadow** |
| 1/18-1/22 | **CLINIC CLOSED**  **MLK DAY** | **CLINIC**  **CLOSED**  **MLK DAY** | | **PPEC Experience** | **RESIDENT**  **LECTURES** | **PCCP Clinic- Care of the chronically ill child**  **CMS Clinic** | **Patient Panel**  **Tower Square**  **Clinic** | **Continuity Clinic**  **CMS Clinic** | **Patient Panel**  **Tower Square**  **Clinic** | **Wealth Management with Financial Consultant** | **Product Knowledge**  **Target** |
| 1/25-1/29 | **BF Clinic**  **Tower Square**  **Clinic** | | **Patient Panel**  **Tower Square**  **Clinic** | **Staffing and Practice Management**  **Tower Square**  **9:00am**  **Nursing Issues**  **Tower Square**  **10:00am** | **RESIDENT**  **LECTURES** | **Magnolia Park- Tour and Therapy Experience**  **Magnolia Park Rehab** | **Patient Panel**  **Tower Square**  **Clinic** | **Continuity Clinic**  **CMS Clinic** | **Adolescent Clinic**  **CMS**  **Clinic** | **Compliance, Billing, and Documentation**  ***PREP Required (print 10 personal pt encounters)** | **Patient Panel**  **CMS**  **Clinic** |
| 2/1-2/5 | **Infant and Toddler Dental Clinic** | **Patient Panel**  **Tower Square**  **Clinic** | | **NBN Nursery-**  **9500**    **Newborn Care & Billing** | **RESIDENT**  **LECTURES** | **ADHD Clinic**  **CMS**  **Clinic** | **Patient Panel**  **Tower Square**  **Clinic** | **Continuity Clinic**  **CMS Clinic** | **Patient Panel**  **Tower Square**  **Clinic** | **Complete TTP final materials (Coding, Proc, & ILP)**  **8:30**  **Tower Square)** | **Interview for job** |

Blue= patient panel Yellow=Special patient panel Orange= CC Pink=Newborn Nursery

Purple= TTP special activity Green= Peds work with attd
