# Supplementary material for: Transition to Pediatric Practice: A Residency Elective Experience to Prepare Senior Pediatric Residents for General Pediatric Primary Care
Source: MedEdPORTAL. 2016 Nov 22;12:10506. doi: 10.15766/mep_2374-8265.10506 (PMC6440492; doi:10.15766/mep_2374-8265.10506)
Supplement: Supplementary file 1 — A. Transition to Pediatric Practice Curriculum Selective Options.docx B. Sample Individualized Learning Plan for Transition to Practice.docx C. Sample Transition to Practice Schedule.docx D. Coding for Pediatrics Presentation.ppt E. RBRVS Presentation.pdf [file mep-12-10506-s001.zip › E. RBRVS Presentation.pdf]

2016 RBRVS
WHAT IS IT AND HOW DOES IT AFFECT PEDIATRICS?

Sanjeev Tuli, MD, MEd
Professor and Associate Chair
Chief, Division of General Pediatrics, UF

RBRVS

- In1992, Centers for Medicare and Medicaid Services (CMS) implemented the “Resource Based Relative Value Scale” physician fee schedule
  - Replaced the Medicare physician payment system of “customary, prevailing, and reasonable” (CPR) charges
  - Derived from the “relative value” of services provided and based on the resources they consume

RBRVS

- The relative value of each service is quantifiable and is based on the concept that there are three components of each service:
  - Amount of physician work that goes into the service
  - Practice expense associated with the service
  - Professional liability expense for the provision of the service

RBRVS

- The relative value of each service is multiplied by Geographic Practice Cost Indices (GPCIs) for each Medicare locality

- Translated into a dollar amount by an annually adjusted conversion factor (CF)
    - 2016 Medicare Conversion Factor = $ 35.80

Physician Work (wRVU)

- ~52% of the total RVUs for each service
- Divided into pre-service, intra-service, and post-service periods that equal the total value of work for each service
  - Physician time required to perform the service
  - Physical effort and technical skill
  - Mental effort and judgment
  - Psychological stress associated with physician’s concern about the iatrogenic risk to the patient

Practice Expense (PE)

- ~44% of the total RVUs for each service
- Beginning in 1998, some CPT codes were assigned two (2) practice expense RVUs:
  - a lesser one for procedures performed in a facility (i.e., a hospital, skilled nursing facility, or ambulatory surgical center)
  - a greater one for procedures/services performed at a non-facility site (i.e., physician's office or patient's home)

Geographic Practice Cost Indices (GPCIs)

- Reflect the relative costs associated with physician work, practice, and professional liability insurance in a Medicare locality compared to the national average relative costs
  - Cost of Living GPCI: Applied to physician work relative values (FL = 1.00)
  - Practice Cost GPCI: Applied to practice expense relative values (Rest of FL = 0.96/ Miami = 1.03)
  - Professional Liability Cost GPCI: Applied to PLI relative values (Rest of FL 1.31/ Ft. Laud. = 1.71/ Miami = 2.49)

Principles of CPT

**Office visit - New**	**99201**	**99202**	**99203**	**99204**	**99205**
Time (minutes)	10	20	30	45	60
Physician work RVU	0.48	0.93	1.42	2.43	3.17
**Office visit - Established**	**99211**	**99212**	**99213**	**99214**	**99215**
Time (minutes)	5	10	15	25	40
Physician work RVU	0.18	0.48	0.97	1.50	2.11
**Consultation – Outpatient**	**99241**	**99242**	**99243**	**99244**	**99245**
Time (minutes)	15	30	40	60	80
Physician work RVU	0.64	1.34	1.88	3.02	3.77

Prolonged Service Cdes – 99354/99355

RBRVS: New patient E/M

- Total Non-facility RVUs for CPT code **99201** (10 min)
  - Work RVUs (0.48) + NFPE RVUs (0.70) + PLI RVUs (0.05)
  - 1.23 x $35.8 = $44.0
- Total Non-facility RVUs for CPT code **99203** (30 min)
  - Work RVUs (1.42) + PE RVUs (1.47) + PLI RVUs (0.15)
  - 3.04 x $35.8 = $108.8
- Total Non-facility RVUs for CPT code **99205** (60 min)
  - Work RVUs (3.17) + PE RVUs (2.36) + PLI RVUs (0.29)
  - 5.82 x $35.8 = $208.4

RBRVS: Outpatient Consultation

- Total Non-Facility RVUs for CPT code **99241**
  - Work RVUs (0.64) + PE RVUs (0.66) + PLI RVUs (0.04)
  - 1.34 x $35.8 = $48.0

- Total Non-Facility RVUs for CPT code **99243**
  - Work RVUs (1.88) + PE RVUs (1.46) + PLI RVUs (0.11)
  - 3.45 x $35.8 = $123.5

- Total Non-Facility RVUs for CPT code **99245**
  - Work RVUs (3.77) + PE RVUs (2.30) + PLI RVUs (0.22)
  - 6.29 x $35.8 = $225.2

RBRVS: Established E/M

- Total non-facility RVUs for CPT code **99211** (5 min)
  - Work RVUs (0.18) + PE RVUs (0.37) +PLI RVUs (0.01)
  - 0.56 x $35.8 = $20.0
- Total non-facility RVUs for CPT code **99213** (15 min)
  - Work RVUs (0.97) + PE RVUs (1.01) +PLI RVUs (0.07)
  - 2.05 x $35.8 = $73.4
- Total non-facility RVUs for CPT code **99215** (40 min)
  - Work RVUs (2.11) + PE RVUs (1.81) + PL I RVUs (0.15)
  - 4.07 x $35.8 = $145.7

RBRVS: New Inpatient

- Total facility RVUs for CPT code **99221** (30 min)
  - Work RVUs (1.92) + PE RVUs (0.75) + PLI RVUs (0.19)
  - 2.86 x $35.8 = $102.4

- Total facility RVUs for CPT code **99222** (50 min)
  - Work RVUs (2.61) + PE RVUs (1.04) + PLI RVUs (0.21)
  - 3.86 x $35.8 = $138.2

- Total facility RVUs for CPT code **99223** (70 min)
  - Work RVUs (3.86) + PE RVUs (1.56) + PLI RVUs (0.29)
  - 5.71 x $35.8 = $204.4

RBRVS: Inpatient Consultation

- Total facility RVUs for CPT code **99251** (35 min)
  - Work RVUs (1.0) + PE RVUs (0.39) + PLI RVUs (0.09)
  - 1.48 x $35.8 = $53.0

- Total facility RVUs for CPT code **99253** (55 min)
  - Work RVUs (2.27) + PE RVUs (0.81) + PLI RVUs (0.18)
  - 3.26 x $35.8 = $116.7

- Total facility RVUs for CPT code **99255** (110 min)
  - Work RVUs (4.0) + PE RVUs (1.66) + PLI RVUs (0.19)
  - 5.85 x $35.8 = $209.5

RBRVS: Critical Care < 28 days

- Total facility RVUs for CPT code **99468**
  - Work RVUs (18.46) + PE RVUs (7.06) + PLI RVUs (1.09)
  - 26.61 x $35.82 = $952.7
- Total facility RVUs for CPT code **99469**
  - Work RVUs (7.99) + PE RVUs (2.74) + PLI RVUs (0.51)
  - 11.24 x $35.8 = $402.4

RBRVS: Critical Care: 29 days-2 Years

- Total facility RVUs for CPT code **99471**
  - Work RVUs (15.98) + PE RVUs (7.1) + PLI RVUs (1.67)
  - 24.75 x $35.8 = $886.1

- Total facility RVUs for CPT code **99472**
  - Work RVUs (7.99) + PE RVUs (2.96) + PLI RVUs (0.61)
  - 11.56 x $35.8 = $413.9

RBRVS: Critical Care: 2 - 5 years

- Total facility RVUs for CPT code **99475**
  - Work RVUs (11.25) + PE RVUs (4.21) + PLI RVUs (0.74)
  - 16.20 x $35.8 = $580.3

- Total facility RVUs for CPT code **99476**
  - Work RVUs (6.75) + PE RVUs (2.55) + PLI RVUs (0.45)
  - 9.75 x $35.8 = $349.2

RBRVS: Critical Care: 6 yrs and older

- Total Facility RVUs - **99291** (30-74 min)
  - Work RVUs (4.50) + PE RVUs (1.42) + PLI RVUs (0.39)
  - 6.31 x $35.8 = $225.9
- Total Facility RVUs - **99292** (each add 30)
  - Work RVUs (2.25) + PE RVUs (1.02) + PLI RVUs (0.19)
  - 3.16 x $35.8 = $113.1

RBRVS: Intensive Care - Infant

- Total facility RVUs for CPT code **99477**
  - Work RVUs (7.00) + PE RVUs (2.68) + PLI RVUs (0.41)
  - 10.09 x $35.8 = $361.2

  - Total facility RVUs for **99478** (< 1500 grams)
    - Work RVUs (2.75) + PE RVUs (.93) + PLI RVUs (0.18)
    - 3.86 x $35.8 = $138.2
  - Total facility RVUs for **99479** (1500-2500 grams)
    - Work RVUs (2.50) + PE RVUs (0.86) + PLI RVUs (0.16)
    - 3.52 x $35.8 = $126.0
  - Total facility RVUs for **99480** (> 2500 grams)
    - Work RVUs (2.40) + PE RVUs (0.82) + PLI RVUs (0.15)
    - 3.37 x $35.8 = $120.6

RBRVS: Sleep Testing (Polysomnography)

- Total Non-Facility RVUs for CPT code **95782** < 6 yrs
  - Work RVUs (2.60) + PE RVUs (26.15) + PLI RVUs (0.29)
  - 29.0 x $35.8 = $1039.7
- Total Non-Facility RVUs for CPT code **95783** (CPAP)
  - Work RVUs (2.83) + PE RVUs (27.34) + PLI RVUs (0.27)
  - 30.44 x $35.8 = $1089.9
- Total Non-Facility RVUs for CPT code **95810** > 6 yrs
  - Work RVUs (2.50) + PE RVUs (14.54) + PLI RVUs (0.21)
  - 17.6 x $35.8 = $630
- Total Non-Facility RVUs for CPT code **95811** (CPAP)
  - Work RVUs (2.60) + PE RVUs (15.68) + PLI RVUs (0.21)
  - 18.49 x $35.8 = $662
